# Development of the Japanese Version of the Test of Self-Conscious Affect-3 (TOSCA-3): A Study among Student and Parent Population

**DOI:** 10.3390/bs14070576

**Published:** 2024-07-07

**Authors:** Toshinori Kitamura, Ayako Hada, Yuriko Usui, Yukiko Ohashi

**Affiliations:** 1 Kitamura Institute of Mental Health Tokyo, 2-26-3 Flat A, Tomigaya, Shibuya-ku, Tokyo 151-0063, Japan; kitamura@institute-of-mental-health.jp (T.K.); yusui@g.ecc.u-tokyo.ac.jp (Y.U.); y-ohashi@jiu.ac.jp (Y.O.); 2 Kitamura KOKORO Clinic Mental Health, 2-26-3 Flat A, Tomigaya, Shibuya-ku, Tokyo 151-0063, Japan; 3 T. and F. Kitamura Foundation for Studies and Skill Advancement in Mental Health, Tokyo 151-0063, Japan; 4 Department of Psychiatry, Graduate School of Medicine, Nagoya University, Nagoya 464-8601, Japan; 5 Department of Community Mental Health and Law, National Institute of Mental Health, National Center of Neurology and Psychiatry, Kodaira, Tokyo 187-8553, Japan; 6 Department of Mental Health and Psychiatric Nursing, Tokyo Medical and Dental University, Bunkyo-ku, Tokyo 113-8510, Japan; 7 Department of Midwifery and Women’s Health, Division of Health Sciences and Nursing, Graduate School of Medicine, The University of Tokyo, Bunkyo-ku, Tokyo 113-0033, Japan; 8 Faculty of Nursing, Josai International University, Togane, Chiba 283-8555, Japan

**Keywords:** self-conscious emotions, factor structure, validity, demographic features

## Abstract

Objective: The Test of Self-Conscious Affect-3 (TOSCA-3) is a scenario-based measure of self-conscious emotions. We aimed to create an abridged version of the TOSCA-3 that is appropriate for Japanese populations and has a good fit with the data, as well as validate its subscales. Methods: The TOSCA-3 was distributed to (a) a university student population (*n* = 512: Study 1) and (b) a parent population (*n* = 260: Study 2). In both studies, items with factor loading < 0.33 were deleted one by one to select culturally appropriate scenarios for each of the six domains of self-conscious emotions. In Study 1, self-conscious emotions were correlated with the other correlates. Results: Most of the final models showed a good fit with the data. In Study 1, the six domains of self-conscious emotions showed correlations with depression and related items, dispositional coping styles, experiences in childhood, ego function, borderline and narcissistic personality traits, and adult attachment styles, almost in the expected fashions. Conclusions: The TOSCA-3 is a useful tool to measure self-conscious emotions among Japanese student and parent populations if a few culturally inappropriate scenarios are deleted.

## 1. Introduction

Emotions play a central role in individual personality and social functioning. Many emotion researchers have attempted to find discrete categorical emotions for over a half-century (e.g., [1,2,3,4]). Kleinginna and Kleinginna [5] attempted to define “emotion” comprehensively. They proposed the following definition:

*Emotion is a complex set of interactions among subjective and objective factors, mediated by neural~hormonal systems, which can (a) give rise to affective experiences such as feelings of arousal, pleasure/displeasure; (b) generate cognitive processes such as emotionally relevant perceptual effects, appraisals, labeling processes; (c) activate widespread physiological adjustments to the arousing conditions; and (d) lead to behavior that is often, but not always, expressive, goal-directed, and adaptive. (p. 355)*

Emotion facilitates motivation and helps a person achieve goals via an extremely complex emotional process. In this process, when we encounter some events, emotions arise in our minds immediately to judge whether they are adaptive or maladaptive. Those emotions are expressed as facial and bodily expressions, essential components of nonverbal communication [1,6]. Those emotions are called basic emotions, including happiness (joy), anger, fear, sadness, disgust, and surprise. Basic emotions guide us to cope and survive [7,8]. 

Self-conscious emotions, on the other hand, are more complex than basic emotions. Since self-conscious emotions require self-awareness and self-representation, they differ from basic emotions [9,10]. The process, which involves self-awareness and self-representation, results in self-evaluations and is followed by the appearance of self-conscious emotions [9,10,11,12,13]. Ideal self-representations, for example, “I want to be a perfect parent” forms identity, and we behave to meet an identity goal in a social and cognitive process. When we come across an event, we appraise the event as relevant to identity goals, generating self-conscious emotions. Both shame and guilt are negative-valence self-conscious emotions. However, there are some differences between the two. Whereas shame is an emotion negatively evaluating the entire self (e.g., “I am incompetent as a parent”), guilt is an emotion evaluating the specific self and own maladaptive behaviour (e.g., “What bad things I have done toward my child!”) [9,10,13,14,15,16,17]. On the other hand, pride is a positive-valence self-conscious emotion with two distinct facets. One is called alpha-pride, which a person experiences in the entire self (e.g., “I am an excellent parent”), and another is called beta-pride, which a person experiences in a specific self (e.g., “I did good things for my child”) [14,15,17]. 

In interpersonal relationships, self-conscious emotions have moral functions because moral standards, moral decisions, and moral behaviours are influenced by self-conscious emotions [15,17]. In addition, pride has the function of promoting the attainment and maintenance of social rank [18].

Externalisation and detachment are responses following the negatively valenced scenarios. Externalisation is a likely defensive manoeuvre in the face of the overwhelming pain of shame. Shame and externalisation involve diametrically opposed attributions in terms of the internal and external. On the other hand, detachment is a response to detaching from incompatible emotional pain [14].

Among instruments to measure self-conscious emotions, the Test of Self-Conscious Affect-3 (TOSCA-3) is unique in that all the items are based on both positive- and negative-valence scenarios. Respondents are given a series of specific, common, day-to-day situations. The TOSCA-3 was examined and supported as a measure of self-conscious emotions in several countries (e.g., shame, guilt, alpha pride, beta pride, externalization, detachment) [19,20,21]. On the other hand, poor agreement with the other shame and guilt proneness scales [22] or gender differences [23] were reported. 

A scenario-based assessment is advantageous in that it is conceptually specific and embedded in a daily context. This is more appropriate and accurate to assess the context-specificity of self-conscious emotions rather than generic questions such as “how often (or much) do you feel ashamed?” It may also be more likely to avoid socially desirable patterns of response [24]. A scenario-based assessment is, however, not without limitations. Tangney [25] noted that compared to adjective checklist measurements, a scenario-based assessment (a) is less reliable, (b) places inevitable constraints on the range of situations, (c) can be confounded by moral standards, and (d) can fail to tap more maladaptive forms of guilt. In addition, we consider that scenario-based approaches are, though accurate to differentiate patterns of self-conscious emotions, very sensitive to cultural norms. Cultural specificity is pivotal in emotion research. For example, a situation presented by the TOSCA-3 is “You are out with friends one evening, and you’re feeling especially witty and attractive. Your best friend’s spouse seems to particularly enjoy your company”. This may be a dinner table shared by two couples. Although this situation may be appropriate to evoke a response of self-conscious emotions in Western cultures, it may not be very much so in other cultures. In Japan, married men usually go out for dinner with male friends, while married women usually go out for lunch with female friends. Sharing a dinner table in a restaurant with two couples is not very common among the Japanese population. We think it feasible to rule out scenarios that are not familiar to Japanese culture to make a Japanese version of the TOSCA-3. This will inevitably be an abridged version of the original instrument.

Another issue is the goodness-of-fit of the factor structure of such an abridged version. The robustness of the factor structure of the original instrument developed and validated in the original cultural background cannot necessarily guarantee the robustness of the abridged version used in a different culture. Also of importance is the effect of common method bias. The TOSCA-3 contains scenarios with positive valence (e.g., “For several days you put off making a difficult phone call. At the last minute you make the call and are able to manipulate the conversation so that all goes well”) and negative valence (e.g., “You make a mistake at work and find out a co-worker is blamed for the error”), it may be subject to a method bias [26]. A mixture of positive- and negative-valence items is likely to lead to careless responses from participants [27,28]. Priming effects may influence the response process because answering initial questions brings information into short-term memory that remains accessible [26]. In such cases, confirmatory factor analysis (CFA) often results in rejection of the one-factor model [29,30]. Therefore, the addition of method factors as a general factor is recommended. 

The present report consists of two parts. One is a secondary analysis of our previous report. It identifies the TOSCA-3 scenarios that are appropriate to Japanese university student populations and have robustness of the factor structure of selected scenarios; the analysis also examines the construct validity of the Japanese version of the TOSCA-3. Another purpose of this study was to examine differences in the TOSCA-3 items between students and parents because of their different interpersonal relationships. 

As measures to identify the scale’s construct validity, we used measures of depression and related items, dispositional coping styles, experiences in childhood, ego function, borderline and narcissistic personality traits, and adult attachment styles. Another data set comes from the data collected from men and women with at least one child (including a foetus) in order to identify the TOSCA-3 scenarios that are appropriate to Japanese parent populations and have a robust factor structure. We used these two different populations on the assumption that the appropriate TOSCA-3 scenario would depend on the demographic characteristics of the participants. It is of great importance in any cultural background that the scenarios for the TOSCA-3 assessment are the same across different demographic populations. This should be supported by the invariance of the factor structure of the measure. Little has been studied from this perspective regarding TOSCA-3. Therefore, in this study, we hypothesised that TOSCA-3 has different constructs, taking the manner of responses to the scenario items in each measure of shame, guilt, alpha-, beta-pride, externalisation, and detachment into account. Our final objective was to select items from TOSCA-3 that are common among students and parents. 

## 2. Study 1

### 2.1. Methods

#### 2.1.1. Study Procedures and Participants

This is a secondary analysis of our data reported previously [31,32,33,34,35,36,37,38,39,40,41,42]. A longitudinal study with a nine-wave, four-month follow-up on various psychological issues was conducted among a convenience sample of students from two universities in Kumamoto, Japan. The students were a mixture of the second to the fourth years. The sample was collected using the convenience sampling method in this study. We assured the participant students’ anonymity in which we asked them only to use a nickname that they created specifically for this study (so that researchers could identify who the participant was). There were 848 eligible students in total. In this report, we used the data of 512 students who attended and responded to the present survey at the 6th wave (when the TOSCA-3 was included in the questionnaire). There were 122 men and 390 women. Their mean (SD) age was 19.5 (2.0) years and 18.9 (1.0) years for men and women, respectively. Some students missed a class over the course of this study period. Therefore, over time, the number of students responding to the survey varied. Every wave was separated by one to two weeks duration, except for the period of four weeks from Wave 7 to Wave 8. The whole study was conducted in 2005.

#### 2.1.2. Measurements

Self-conscious emotions: We used the TOSCA-3 [43]. This is a self-report measure of six self-conscious emotions: shame, guilt, alpha- and beta-prides, externalisation, and detachment. The TOSCA-3 contains eleven negative- and five positive-valence scenarios, with four or five responses reflecting one of the six self-conscious affects. Each response is given (e.g., “You make plans to meet a friend for lunch. At 5 o’clock, you realize you stood him up”). Each scenario is followed by responses representing brief descriptions of shame, guilt, alpha- and beta-prides, externalisation, and detachment (unconcern) (e.g., for shame, one would think: “I’m inconsiderate”; for guilt, one would think: “I should make it up to him as soon as possible”). Those responses were rated on a 5-point scale. This was translated into Japanese with permission from the original author, with verification via retranslation into English.

Social desirability: We used the Japanese version [44] of the Social Desirability Scale [45]. This is a self-report to measure the tendency to respond to a question in a socially desirable fashion. The Japanese version consists of ten items scored on a 5-point scale. Higher scores indicate a social desirability tendency. This was distributed to the participants at Wave 5.

Depression: We used seven items of the Self-Rating Depression Scale (SDS; [46]) that belonged to the affective category of the scale [47]. The SDS is a widely used 20-item self-report measure of state depression. Each item was rated on a 4-point scale. Its 3-factor structure was reported for a Japanese university student population: affective, cognitive, and somatic [47]. The SDS was given at every wave.

Suicidality: We used an item from the SDS: “I feel that others would be better off if I were dead”. This was assessed on a 4-point scale. This was given at every wave.

Thinking error: We used the Thinking Error Scale (TES: [48]) to measure cognitive distortion. The original TES consists of 19 items. We selected six items with the highest factor loadings in Tanno et al.’s study [48]. Each item was rated on a 4-point scale. The TES was given at each wave.

Automatic thought: We used the Japanese version [49] of the Automatic Thoughts Questionnaire-Revised (ATQ-R; [50]) to assess the extent to which an individual experiences negative automatic thought. The ATQ-R was based on the Automatic Thought Questionnaire [51], widely used in different languages such as Norwegian [52], Turkish [53], and Korean [54]. The original ATQ-40 consists of 40 items on a 5-point Likert scale. The Japanese version of the ATQ-R was back-translated into English to confirm that the translation was consistent with the original intent. Examples of the items include “No one understands me” and “Why can’t I ever succeed?” In the present study, we selected six items with the highest factor loadings in Kendall et al.’s study [50]. The ATQ-R was given at each wave.

Dispositional coping style: We used the Japanese version [55] of the Coping Inventory for Stressful Situations (CISS; [56]) to assess dispositional coping styles. The CISS consists of 48 items on a 5-point scale. The CISS’s three subcategories are task-oriented, emotion-oriented, and avoidance-oriented coping. Task-oriented coping is usually adaptive, taking into consideration priorities and carefully selecting a course of action. Emotion-oriented coping is less adaptive, blaming oneself about the events and becoming preoccupied with worries and anxieties. Avoidance-oriented coping is another non-adaptive way of dealing with the situation. People participate in non-problem-solving behaviours as a way of ignoring the problem. The CISS was distributed to the participants at Wave 1.

Perceived rearing during childhood: We used the Japanese version [57,58] of the Parental Bonding Instrument (PBI; [59]) to measure perceptions of parental attitudes towards the participant as a child. Each item is scored on a 4-point scale. It consists of two subcategories: care (12 items) and overprotection (13 items). The PBI was given at Wave 3.

Child abuse and neglect: We used the Japanese version [34] of the Child Abuse and Trauma Scale (CATS: [60]) to assess the traumatic experiences in childhood. It consists of 38 items on a 5-point scale. A five-factor structure for the Japanese version was reported [33]. This resulted in five subscales: (1) neglect and emotional abuse (14 items), (2) punishment and scolding (10 items), (3) sexual maltreatment (6 items), (4) authoritarianism (5 items), and (5) marital disharmony (3 items). The CATS was given at Wave 3.

Resilience: We used the Japanese version [31] of the Resilience Scale (RS; [61]) to assess resilience. The original RS consists of 25 items on a 7-point scale. We modified the number of choices to five in order to adjust the number of choices to match most of the other questionnaires in this study. The RS was given at Wave 4.

Self-efficacy: We used the Japanese version [62] of the Self-Efficacy Scale (SES; [63]) to assess general self-efficacy proposed by Badura [64,65,66]. The Japanese version of the SES is comprised of 23 items on a 5-point scale. A higher score indicates a greater perception of self-efficacy. The Japanese version was reported as having good validity and reliability [62].

Borderline personality traits: We used the Japanese version [67] of the Inventory of Personality Organisation (IPO; [68]) to assess borderline personality traits. The IPO consists of 83 items on a 5-point scale. Based on the central dimension of Kernberg’s [69] personality organisation model, it has three primary dimensions: primitive defences (16 items), identity diffusion (21 items), and reality testing (20 items). There are two additional scales: aggression (18 items) and moral values (8 items with two primitive defences items and one identity diffusion item). The IPO was given at Wave 7.

Narcissistic personality trait: We used the Japanese version [70] of the Narcissistic Personality Inventory (NPI; [71]) to assess narcissistic traits. The original NPI consisted of 233 items being divided into two forms. A short version of 54 items was proposed by Emmons [72]. Oshio [73] proposed an 18-item Japanese version (NPI-S). The NPI-S has three subcategories: feeling superior (6 items), desire for admiration (6 items), and assertiveness (6 items). The NPI-S was rated on a 5-point scale. The NPI-S was distributed to participants at Wave 5.

Adult attachment style: We used the Japanese version [74] of the Adult Attachment Relationship Questionnaire (RQ; [75]) to assess the four categories of adult attachment (secure, fearful, preoccupied, and dismissing). The RQ has four paragraphs, describing each attachment style. The participants were asked to rate the extent to which each description would correspond to their relationship with their intimate person on a 7-point scale. Its reliability (Bartholomew and Horowiz, 1991) and validity [76] were reported as good. The RS was distributed to participants at Wave 5.

#### 2.1.3. Data Analysis

If an item behaves statistically heterogeneously among scale items, it is reasonable to assume that the item is inappropriate. To determine if a scenario is inappropriate, we examined each subscale as a single item set. Because there were a few missing cases for the TOSCA-3 items, we examined whether the data were missing completely at random (MCAR) via Little’s MCAR test. After calculating the mean, SD, skewness, and kurtosis of all the items of the six TOSCA-3 domains, we performed a single-factor exploratory factor analysis (EFA) of the items of each domain, separately. In order to examine whether the data set was appropriate for EFA, we performed the Keiser–Meyer–Olkin (KMO) index and Bartlett’s sphericity test [77]. An item of the lowest factor loading was deleted before repeating an EFA, and this procedure was reiterated until no items had a factor loading < 0.33 [78]. We selected the scenario for which all the items had factor loading > 0.33 in its domain. The items of each domain selected in such a manner were subjected to a confirmatory factor analysis (CFA). Goodness-of-fit was examined by chi-squared (*χ*^2^), comparative fit index (CFI), root mean square error appropriation (RMSEA), and Akaike information criteria (AIC). A good fit was defined as chi-squared divided by degrees of freedom (*χ*^2^/*df)* < 2, CFI > 0.97, and RMSEA < 0.05. An acceptable fit was defined as *χ*^2^/*df* < 3, CFI > 0.95, and RMSEA < 0.08 [79,80]. If the goodness-of-fit of a selected model did not reach a satisfactory level, we tried a bifactor model [81,82] with two specific factors determined by the positive and negative valences of the scenarios. It is of note that because the two types of pride and detached domains had either positive or negative valence in scenarios, bifactor models were inappropriate. Whenever a bifactor model was selected, omega indices, explained common variance (ECV), and percent of uncontaminated correlations (PUC) were calculated in order to identify the degree of unidimensionality of the model. ECV is a proportion of all common variance explained by the general factor. ECV is provided by the following equation:

$$\mathrm{ECV}=\frac{\sum \lambda^{2}Gen}{\sum \lambda^{2}Gen+\sum \lambda^{2}Group\;1+\cdots +\sum \lambda^{2}Group\;k\;}$$

where *Gen* is the general factor and *Group 1* to *Group k* are 1 to k group factors [83,84,85,86]. PUC provides the proportion of elements of the covariance matrix that are only modelled by the general factor. PUC is given by the following equation:

$$\mathrm{PUC}=\frac{\frac{n_{items}(n_{items}-1)}{2}-n_{groups}\times \frac{n_{ipg}(n_{ipg}-1)}{2}}{\frac{n_{items}(n_{items}-1)}{2}}$$

where *n_items* is the total number of items, *n_groups* is the number of group factors, and *n_ipg* is the number of items per group factor [83]. Relative bias is defined as the ratio of the difference between theoretical and estimated coefficients to the theoretical coefficient [83]. ECV predicts a relative bias in the structural coefficient, and PUC moderates this relationship [83,85]. When PUC > 0.80 or when ECV > 0.60 and *omega hierarchicals (ωH)* > 0.7, relative bias by using a unidimensional measurement model rather than a bifactor measurement model is likely to be slight [85].

We calculated the subscales of the Japanese TOSCA-3 by adding the raw scores of the items that remained in each self-conscious domain. These subscale scores were correlated with the other scale scores. The data were analysed using IBM SPSS Statistics for Windows (version 24, IBM Corp., Tokyo, Japan).

### 2.2. Results

Little’s MCAR test was *χ*^2^ = 1170.712, *df* = 1120, and *p* = 0.142. This means that MCAR was not rejected. Therefore, we performed the subsequent analyses dealing with missing values casewisely.

Almost all the TOSCA-3 items had skewness < 2 and kurtosis < 4 (Table 1, Table 2, Table 3, Table 4, Table 5 and Table 6). After deleting items for which factor loadings were less than 0.33, all six TOSCA-3 domains except for shame and detachment showed a good fit with the data (Table 7).

Then we correlated the scores of each TOSCA-3 domain with the other variables (Table 8). None of the domain scores were correlated with the Social Desirability Scale scores. The scores of shame were correlated with depression, suicidal thought, thinking error, and automatic thought, whereas the scores of detachment were negatively correlated with depression, suicidal thought, and thinking error. Shame, guilt, and externalisation were correlated with emotion-oriented coping style, whereas only guilt was correlated with task-oriented coping style. Of the childhood experiences, shame was positively associated with neglect and emotional abuse, whereas guilt was associated with fewer experiences of sexual maltreatment and parental authoritarianism. Both resilience and self-efficacy were lower with students high in shame. The two types of pride were associated with resilience. Both shame and alpha-pride were associated with some subscales of borderline personality traits, but it was only the two pride scores that were correlated with narcissistic personality traits. Finally, self-image but not other-image in adult attachment was negatively correlated with shame and detachment.

### 2.3. Discussion

Although the abridged version of the Japanese TOSCA-3 showed acceptable goodness-of-fit with the data as well as no significant correlations with social desirability for almost all the domains, some scenarios seemed inappropriate to measure self-conscious emotions among a Japanese university student population. Scenario 11 was found to be inappropriate as a means to measure shame, guilt, and externalisation. This situation is “You and a group of co-workers worked very hard on a project. Our boss singles you out for a bonus because the project was such a success”. Because our participants were all students and young, they were not familiar with a workplace situation like this. Scenarios 3, 5, and 16 were inappropriate as a means to measure shame. In Scenario 3, the participants may find it difficult to imagine a situation in which they and their partner go out to dinner with a friend couple. Scenario 5 was another workplace situation that students may find it difficult to imagine. Scenario 16 presents a situation in which the participant is invited to a housewarming party with a glass of red wine. A housewarming party is not a typical event in Japan and students typically drink beer (that does not stain a cream-coloured carpet) rather than wine. Scenario 8 was found to be inappropriate as a means to measure beta-pride. Scenario 8 presents a situation where participants borrow money a few times but pay it back quickly. In this situation, Japanese students would perceive it as natural to pay back the money as soon as possible. Therefore, they are unlikely to feel pride. Scenario 15 was inappropriate for externalisation and detachment. Here, the participant was asked to imagine a situation where he/she is asked to look after his/her friend’s dog. Again, this is not a usual situation in Japan. Scenario-based measures of self-conscious emotions have advantages. However, our results warn researchers to exercise caution when selecting scenarios to suit the culture specific features of the target populations.

In our study, TOSCA-3 shame was linked to depression and suicidality. This is in line with the report that shame is linked to depression [16]. Some past investigations reported a link between guilt and depression [87,88,89,90,91]. Nevertheless, these previous reports paid little attention to the difference between guilt and shame. The link of depression was in many studies equal between shame and guilt [92,93] whereas Tangney et al. [16] emphasised the link of Shame with depression. Our results added a new finding that shame was correlated not only to depression but also to cognitive distortion and the tendency of automatic thought that are theoretically related to depression.

Both shame and guilt were linked to emotion-oriented coping styles, but it was only guilt that was linked to task-oriented coping styles. Guilt may prompt one to undo the damage one caused so that they are more likely to adopt problem-focused coping behaviours. This is in line with the stronger association between shame and depression than guilt and depression. Thus, task-oriented coping style attached to guilt may protect individuals who are exposed to stressful life events from developing depression.

Our study indicated that, whereas TOSCA-3 shame was linked to childhood neglect and emotional abuse, TOSCA-3 guilt was linked to less sexual maltreatment and parental authoritarianism. This is in contrast to Bennett et al. [94] and Stuewig and McCloskey [95], who reported that shame proneness was linked to the experiences of physical abuse. This difference may be due to the age of the participants. Ours were adolescents and young adults, whereas Bennett et al.’s [94] and Stuewig and McCloskey’s [95] were children. Our students’ shame or guilt were not linked to perceived rearing, including low care or overprotection.

In our findings, unlike guilt, shame was associated with maladjusting personality traits such as low resilience and self-efficacy and higher borderline personality traits, as well as lower narcissistic personality traits. In contrast, alpha-pride was associated with resilience, primitive defence, and the three subcategories of narcissistic personality traits. This suggests that although shame and alpha-pride share their association with borderline personality traits, only alpha-pride is linked to narcissistic personality traits. In this point of view, our finding was not consistent with the literature, which suggested associations between shame, alpha-pride, and narcissistic personality traits only [10,96].

Self-image of adult attachment style was poorer among students high in Shame. This was not the case with the other self-conscious emotions. Detachment was linked to a better self-image. Taking into consideration the association of detachment with lower depression, suicidality, and thinking error, we presume that students high in detachment have better ego function and are more resilient to stressful situations.

Our results indicated that, among the self-conscious emotions, shame was clearly associated with poorer mental health and maladaptive personality traits. Guilt showed, though sharing common correlates with shame in some areas, a clear distinction from shame.

## 3. Study 2

As we mentioned in the Introduction, the TOSCA-3 is a scenario-based, widely used questionnaire. As its items reflect characteristics of a unique culture, psychometric properties may vary depending on cultural aspects of the target population. In Study 2, we re-examined the culture specificity of the TOSCA-3 in a Japanese parent population, where different items may be selected for scenarios for the TOSCA-3.

### 3.1. Methods

#### 3.1.1. Measurement

Self-conscious emotions: The TOSCA-3 was used; however, phrases in the scenarios and multiple choices were slightly modified by TK and AH to be suitable for current Japanese expressions.

#### 3.1.2. Study Procedures and Participants

The sample came from our internet survey to validate the Scale of Parent-to-Child Emotions [97]. The participants, consisting of 780 fathers and 780 mothers with at least one child (including a foetus), were allocated into 6 groups to make homogenous samples across groups in terms of parental gender and age stage of their child. This survey was conducted with the cooperation of Cross Marketing Inc. (Shinjuku, Tokyo) in 2022. They have several research panels to collect samples, and a web questionnaire was delivered via e-mail to the premise-targeted people. In this study, 729,559 people were estimated to be first recruit premise targets across demographic features (i.e., gender, and child’s age) within their panels for screening questionnaires. The response rate was 9.23–13.23%. The survey consisted of 6 groups allocated with different questionnaires. One of those included the TOSCA-3 as an external variable. In Study 2, we used the data from this group (*n* = 260). Men and women were equal in number, and their mean (SD) age was 39.2 (9.6) and 34.3 (8.3) years for men and women, respectively. Other demographic characteristics of participants in Study 2 are shown in Supplementary Table S1. Anonymity was assured, and all responses were voluntary.

#### 3.1.3. Data Analysis

Data analysis followed and replicated the procedures used in Study 1 to identify the factor structure of the TOSCA-3. The data were analysed using IBM SPSS Statistics for Windows (version 29, IBM Corp.).

### 3.2. Results

Almost all the TOSCA-3 items had skewness < 2.0 and kurtosis < 4.0 (Table 9, Table 10, Table 11, Table 12, Table 13 and Table 14). Items with factor loading < 0.33 were deleted by each domain. Bifactor models (with method factors) improved the goodness-of-fit for shame, guilt, and externalisation but it failed to reach CFI = 0.95 for shame and externalisation (Table 15). One-factor models for beta-pride and detached showed unsatisfactory goodness-of-fit. Those models’ CFI were 0.879 and 0.881, respectively, after deleting items where factor loadings were less than 0.33. Because beta-pride and detached domains had either positive or negative valence scenarios, bifactor models were inappropriate.

Omega indices, ECV, and PUC for the shame, guilt, and externalisation domains were calculated to determine whether monodimensionality would override multidimensionality in a bifactor model (Table 16). Whereas all PUCs were lower than 0.80, all ECVs were higher than 0.60, and all *ωH* were higher than 0.80. When PUC > 0.80 or when ECV > 0.60 and *ωH* > 0.7, relative bias by using a unidimensional measurement model is likely to be slight [85]. Therefore, the shame, guilt, and externalisation domains in TOSCA-3 were proven to be unidimensional. Coefficient omegas for alpha pride, beta pride, and detachment domains were 0.65, 0.60, and 0.78, respectively. They were calculated as positing zero factor loading of the (invisible) general factor.

### 3.3. Discussion

In Study 2, although all domains of the abridged version of the Japanese TOSCA-3 were suggested to be unidimensional, goodness-of-fit did not reach a satisfactory level for a few domains. Some scenarios or choices seemed inappropriate to measure self-conscious emotions among Japanese parents. As in Study 1, Scenario 3 was not appropriate for measuring shame among parents. This scenario is a dinner table shared by two couples. There may be a few occasions for a dinner outing with a friend or couple in Japanese culture. Cultural differences may deserve consideration. Americans may feel stronger shame in situations in which others point out their personal flaws, whereas Japanese elicit shame more in situations in which they themselves realize failure at maintaining “face” in public [98]. The Japanese sense of guilt also ties with one’s awareness of another as a victim of one’s action or omission [99]. Because the choice phrases of Scenario 3 are private concerns, they may not have the ability to discriminate against shame or guilt among the Japanese population. Scenario 5 was also inappropriate to measure shame and detached. The statement of scenario 5 is “You make a mistake at work and find out a co-worker is blamed for the error”. Shame implies an egocentric concern, whereas guilt is a more allocentric concern for the consequences of one’s other-oriented behaviour in the Japanese sense [100]. Therefore, Japanese people may feel more guilt than shame when their co-worker (instead of themselves) is blamed by someone.

Scenario 8 is inappropriate to measure beta-pride. The choice of beta-pride is “You would be proud that you repaid your debts” for Scenario 8. Beta-pride is feelings of pride stemming from evaluations of a specific behaviour [14,15,17]. Beta-pride depends on settings for a specific behaviour. The kinds of behaviours people feel pride towards may differ depending on individual cultural settings.

## 4. General Discussion

Both Studies 1 and 2 showed that some TOSCA-3 scenarios were inappropriate as a measure of self-conscious emotions among Japanese populations. There were scenarios that were found inappropriate for both student and parent populations. Thus, Scenarios 3 and 5 were not appropriate as scenes for shame assessment, Scenario 6 was inappropriate as a scene for guilt assessment, Scenario 8 was inappropriate as a scene for beta-pride assessment, Scenarios 4 and 11 were inappropriate as scenes for externalisation assessment, and Scenario 5 was inappropriate as a scene for detachment assessment. These scenarios, as noted earlier, provide situations that are not very familiar to Japanese students and parents. Scenario 7 was not appropriate for a shame assessment only among parents, while Scenario 16 was not appropriate for shame assessment only for students.

Detachment is a response to negative situations with an attitude involving little personal investment [101]. A detached response may not arise when someone intimate is to be blamed. In situations where a co-worker is blamed, Japanese people may respond emotionally by being empathetic. Shame, guilt, and detachment in other scenarios can be explained by the same theoretical frame: egocentric or allocentric.

Externalisation is a defensive manoeuvre against the overwhelming pain of shame experiences [15]. However, the externalisation of blame may lead to difficulty in interpersonal relationships. There is a sense of values in close relationships that are based on harmony in Japanese culture [102]. Therefore, externalisation may arise in different situations between populations with backgrounds of Western and Japanese cultures.

### 4.1. Practice Implications and Further Research

Our results warn researchers when using the TOSCA-3 in a Japanese population that because scenarios are culture/subculture specific, we should be cautious in selecting scenarios suitable for the target population. Other than this, the TOSCA-3 can capture self-conscious emotions consisting of several constructs effectively. Its construct validity showed that the TOSCA-3 may be a useful tool to assess self-conscious emotions among Japanese students and parents. The TOSCA-3 can be used to test the convergent validity of the newly developed scales that measure neighbouring concepts.

Study 1 of our report provided evidence of construct validity of self-conscious emotion domains. Although shame and guilt are both self-blaming emotions, they showed different associations with current mood states, personality traits, dispositional coping styles, adult attachment, and childhood experiences. Both alpha- and beta-prides were associated with task- and avoidance-oriented coping styles, resilience, and narcissistic personality traits, but they differ in terms of borderline personality traits in that only alpha-pride was characterised by high primitive defence and moral value. Although associations between alpha-pride and narcissistic personality traits were previously suggested in the literature [18], alpha-pride was also associated with borderline personality. This finding should take further research into account.

### 4.2. Limitations

There are several limitations to our research. First, Study 1 was a secondary analysis of the sample study, which was conducted in 2005. As the time and cultural contexts were different between 2005 and now, the emotional state of the students may differ. Second, this study was not a comparison of samples representing actual populations from different countries. Further research is needed to identify situations in which feelings of self-conscious emotions are evoked by different cultural backgrounds, across countries.

## 5. Conclusions

Our Studies 1 and 2 demonstrated that the TOSCA-3 is a useful tool to measure self-conscious emotions among Japanese student and parent populations if a few culturally inappropriate scenarios were deleted. Our finding suggested that appropriate scenarios for different populations should be used for clinical situations and research, in TOSCA-3.

## Figures and Tables

**Table 1:** Mean, SD, skewness, kurtosis, and factor loading of the original and final EFA of TOSCA-3 shame domain items (Study 1).

Scenario Number and Choice	*n*	Mean	*SD*	Skewness	Kurtosis	EFA Factor Loading	
						Original	Final
01A	508	3.77	1.21	−0.8	−0.3	0.49	0.48
02B	511	2.32	1.19	0.5	−0.7	0.38	0.36
03E	508	2.93	1.28	0.0	−0.9	0.22	---
04A	511	3.94	1.14	−1.0	0.2	0.58	0.57
05C	510	1.80	0.96	1.1	0.5	−0.06	---
06C	510	3.55	1.13	−0.5	−0.5	0.46	0.44
07A	509	2.06	1.12	0.8	−0.3	0.36	0.34
08A	508	3.40	1.30	−0.4	−1.0	0.41	0.41
09B	506	3.69	1.17	−0.6	−0.5	0.61	0.63
10D	506	3.99	1.09	−1.0	0.4	0.54	0.55
11B	506	2.59	1.17	0.2	−0.9	0.28	---
12B	506	2.99	1.18	−0.1	−0.8	0.45	0.44
13B	506	3.96	1.01	−1.0	0.8	0.60	0.60
14A	506	3.74	1.06	−0.7	−0.1	0.56	0.58
15A	506	3.93	1.14	−1.0	0.2	0.63	0.65
16C	506	2.87	1.32	0.0	−1.1	0.32	---

Note. KMO = 0.858; Bartlett’s test of sphericity, *χ^2^* (*df*) = 1442.960 (120) (*p* < 0.001) by original items. KMO = 0.869; Bartlett’s test of sphericity, *χ^2^* (*df*) = 1171.143 (66) (*p* < 0.001) by final items.

**Table 2:** Mean, SD, skewness, kurtosis, and factor loading of the original and final EFA of TOSCA-3 guilt domain items (Study 1).

Scenario Number and Choice	*n*	Mean	*SD*	Skewness	Kurtosis	EFA Factor Loading	
						Original	Final
01C	510	4.26	1.05	−1.5	1.5	0.43	0.42
02A	511	4.29	0.99	−1.6	2.2	0.43	0.43
03A	509	3.58	1.17	−0.6	−0.4	0.37	0.37
04C	510	4.00	1.00	−0.9	0.4	0.48	0.47
05D	510	4.11	0.95	−1.0	0.8	0.53	0.54
06B	510	3.05	1.28	−0.1	−1.1	0.24	---
07D	510	4.51	0.76	−1.8	3.6	0.57	0.57
08C	507	4.36	0.84	−1.6	2.7	0.66	0.66
09D	505	4.31	0.93	−1.4	1.8	0.65	0.65
10C	506	4.46	0.84	−1.8	3.5	0.67	0.67
11E	506	2.79	1.18	0.2	−0.7	0.20	---
12D	506	3.22	1.12	−0.2	−0.5	0.36	0.36
13C	506	4.12	0.94	−1.0	0.6	0.62	0.62
14C	506	3.56	1.09	−0.5	−0.3	0.49	0.49
15C	506	4.34	0.90	−1.5	2.1	0.67	0.67
16B	505	4.17	1.07	−1.2	0.7	0.61	0.60

Note. KMO = 0.917; Bartlett’s test of sphericity, *χ^2^* (*df)* = 1798.433 (120) (*p* < 0.001) by original items. KMO = 0.924; Bartlett’s test of sphericity, *χ^2^* (*df*) = 1696.735 (91) (*p* < 0.001) by final items.

**Table 3:** Mean, SD, skewness, kurtosis, and factor loading of the original and final EFA of TOSCA-3 alpha-pride domain items (Study 1).

Scenario Number and Choice	*n*	Mean	*SD*	Skewness	Kurtosis	EFA	
						Original	Final
03B	509	2.97	1.13	−0.0	−0.7	0.61	0.61
06A	510	3.38	1.11	−0.4	−0.5	0.45	0.45
08D	507	2.62	1.05	0.2	−0.4	0.35	0.35
11D	506	3.25	1.05	−0.3	−0.4	0.62	0.62
14E	505	3.43	1.18	−0.4	−0.5	0.37	0.37

Note. KMO = 0.716; Bartlett’s test of sphericity, *χ^2^* (*df*) *=* 220.831 (10) (*p* < 0.001) by original (final) items.

**Table 4:** Mean, SD, skewness, kurtosis, and factor loading of the original and final EFA of TOSCA-3 beta-pride domain items (Study 1).

Scenario Number and Choice	*n*	Mean	*SD*	Skewness	Kurtosis	EFA	
						Original	Final
03C	509	3.54	1.10	−0.6	−0.2	0.61	0.61
06D	510	2.62	1.14	0.2	−0.7	0.36	0.33
08E	507	2.39	1.22	0.5	−0.7	0.24	---
11C	506	3.92	1.00	−0.9	0.5	0.46	0.49
14D	506	3.71	1.10	−0.6	−0.3	0.56	0.55

Note. KMO = 0.649; Bartlett’s test of sphericity, *χ^2^* (*df*) = 198.148 (10) (*p* < 0.001) by original items. KMO = 0.657; Bartlett’s test of sphericity, *χ^2^* (*df)* = 162.557 (6) *(p* < 0.001) by final items.

**Table 5:** Mean, SD, skewness, kurtosis, and factor loading of the original and final EFA of TOSCA-3 externalisation domain items (Study 1).

Scenario Number and Choice	*n*	Mean	*SD*	Skewness	Kurtosis	EFA	
						Original	Final
01D	508	1.91	1.06	1.0	0.2	0.45	0.47
02C	511	1.89	1.10	1.1	0.2	0.57	0.61
03D	508	2.94	1.16	−0.1	−0.7	0.30	---
04B	509	2.57	1.13	0.3	−0.7	0.30	---
05A	510	1.84	1.00	1.2	0.9	0.42	0.42
06E	510	2.54	1.07	0.2	−0.5	0.41	0.44
07B	510	1.68	1.01	1.5	1.3	0.48	0.49
08B	507	1.97	0.92	0.7	−0.0	0.46	0.45
09A	506	2.01	1.12	0.8	−0.5	0.46	0.46
10B	506	1.85	0.99	1.1	0.8	0.47	0.48
11A	505	2.46	1.01	0.1	−0.4	0.34	---
12C	506	2.36	1.12	0.5	−0.5	0.32	---
13A	506	2.73	1.16	0.1	−0.7	0.41	0.36
14B	505	2.46	1.02	0.3	−0.5	0.35	0.35
15B	506	2.51	1.36	0.5	−1.0	0.34	---
16D	505	1.69	1.00	1.4	1.5	0.46	0.43

Note. KMO = 0.841; Bartlett’s test of sphericity, *χ^2^* (*df*) = 871.819 (120) (*p* < 0.001) by original items. KMO = 0.852; Bartlett’s test of sphericity, *χ^2^* (*df*) = 653.852 (55) (*p* < 0.001) by final items.

**Table 6:** Mean, SD, skewness, kurtosis, and factor loading of the original and final EFA of TOSCA-3 detachment domain items (Study 1).

Scenario Number and Choice	*n*	Mean	*SD*	Skewness	Kurtosis	EFA	
						Original	Final
01B	509	2.82	1.13	0.1	−0.9	0.26	---
02D	511	2.57	1.20	0.2	−0.9	0.56	0.54
04D	510	2.76	1.23	0.2	−1.0	0.51	0.52
05B	510	2.83	1.27	0.0	−1.1	0.16	---
07C	510	2.77	1.34	0.1	−1.2	0.59	0.59
09C	506	2.98	1.21	−0.2	−0.9	0.57	0.59
10A	506	2.30	1.23	0.6	−0.7	0.44	0.43
12A	506	3.31	1.13	−0.2	−0.8	0.23	---
13D	506	3.17	1.14	−0.1	−0.7	0.62	0.61
15D	505	1.48	0.85	1.9	3.7	0.30	---
16A	506	2.17	1.20	0.8	−0.4	0.35	---

Note. KMO = 0.784; Bartlett’s test of sphericity, *χ^2^* (*df*) = 709.186 (55) (*p* < 0.001) by original items. KMO = 0.778; Bartlett’s test of sphericity, *χ^2^* (*df*) = 502.209 (15) (*p* < 0.001) by final items.

**Table 7:** Factor structure models of TOSCA-3 self-conscious emotion domains (Study 1).

Model	χ^2^	*df*	χ^2^/*df*	Δχ^2^ (*df*)	CFA	ΔCFI	RMSEA	ΔRMSEA	AIC
**Shame**									
1-factor	148.197	54	2.744	Ref	0.916	Ref	0.058	Ref	220.197
With method factor +									
**Guilt**									
1-factor	98.547	65	1.516	Ref	0.978		0.032		176.547
With method factor +									
**Alpha-pride**									
1-factor	3.096	5	0.619	Ref	1.000		0.000	Ref	33.096
**Beta-pride**									
1-factor	5.506	2	2.753	Ref	0.977	Ref	0.059		29.506
**Externalisation**									
1-factor	53.719	44	1.221	Ref	0.983	Ref	0.021	Ref	119.719
With method factor	26.662	32	0.833	27.057 (12) **	1.000		0.000		116.662
**Detachment**									
1-factor	52.210	9	5.801	Ref	0.910	Ref	0.097	Ref	88.210

Note. + improper solution: ** *p* < 0.01; CFI, comparative fit index; RMSEA, root mean square error approximation; AIC, Akaike information criterion.

**Table 8:** Correlations of the TOSCA-3 domains with other variables (Study 1).

	Wave	Shame	Guilt	Alpha Pride	Beta Pride	Externali-Sation	Detachment
*rersponse bias* (*n* = 452)							
Social Desirablity Scale	5	−0.05	0.07	−0.00	−0.02	−0.08	0.05
*depression and correlates* (*n* = 512)							
Depression	6	0.30 ***	0.11 *	−0.05	−0.10 *	0.09 *	−0.16 ***
Suicidal thought	6	0.25 ***	0.04	−0.09 *	−0.11 *	0.02	−0.18 ***
Negative life events	6	0.16 **	0.08	−0.01	−0.05	0.07	−0.13
Thinking error	6	0.39 ***	0.16 **	−0.01	−0.01	0.08	−0.17 ***
Automatic thought	6	0.27 ***	0.01	−0.08	−0.12 *	0.09 *	−0.06
*Dispositional coping styles* (*n* = 431)							
Task-oriented coping	1	0.05	0.21 ***	0.24 ***	0.14 **	0.11 *	0.12 *
Emotion-oriented coping	1	0.41 ***	0.19 ***	0.05	0.01	0.17 ***	−0.13 **
Avoidance-oriented coping	1	0.09	0.16 **	0.19 ***	0.21 ***	0.11 *	0.13 **
*childhood experinces: PBI* (*n* = 450)							
Father’s care	3	−0.03	0.12 *	0.03	0.09	−0.09	0.00
Father’s overprotection	3	−0.01	−0.11	0.03	−0.08	0.18 ***	0.03
Mother’s care	3	−0.03	0.08	0.09	0.13 *	−0.06	0.04
Mother’s overprotection	3	0.07	−0.11 *	−0.02	−0.10	0.17 **	0.10
*childhood experinces CATS* (*n* = 441)							
Neglect and emotional abuse	2	0.18 ***	0.09	0.06	−0.01	0.03	−0.04
Punishiment and scolding	2	0.11 *	0.06	0.08	−0.01	0.06	0.03
Sexual maltreatment	2	−0.11 *	−0.19 ***	−0.01	−0.05	−0.12 *	0.07
Authoritarianism	2	−0.06	−0.21 ***	−0.06	−0.10 *	0.14 **	−0.00
Marital dysharmony	2	−0.01	−0.06	0.08	0.03	0.06	0.05
*ego function* (*n* = 447)							
Resilience	4	−0.24 ***	0.10 *	0.25 ***	0.18 ***	−0.03	0.15 **
Self-efficacy	4	−0.23 ***	0.05	0.11 *	0.11 *	−0.14 **	0.00
*boderline personality* (*n* = 435)							
Primitive defence	7	0.33 ***	0.09	0.17 ***	0.04	0.24 ***	−0.00
Identity difusion	7	0.37 ***	0.17 ***	0.15 **	0.04	0.12 *	−0.06
Reality testing	7	0.23 ***	0.00	0.08	−0.04 *	0.20 ***	−0.04
Aggression	7	0.12 *	−0.14 **	0.09	−0.03	0.28 ***	0.02
Moral values	7	0.21 ***	−0.03	0.17 ***	0.06	0.27 ***	0.05
*narisisitic personality* (*n* = 452)							
Feeling superior	5	−0.17 ***	−0.02	0.35 ***	0.19 ***	0.19 ***	0.21 ***
Desire for admiration	5	0.11 *	0.14 **	0.34 ***	0.26 ***	0.12 **	0.04
Assertiveness	5	−0.16 **	0.09	0.23 ***	0.19 ***	0.03	0.16 **
*adult attachment* (*n* = 452)							
Self image	5	−0.25 ***	−0.06	0.12 *	0.08	0.00	0.18 ***
Other-image	5	−0.05	0.07	0.01	0.07	−0.04	−0.02

Note. * *p* < 0.05; ** *p* < 0.01, *** *p* < 0.001.

**Table 9:** Mean, SD, skewness, kurtosis, and factor loading of the original and final EFA of TOSCA-3 shame domain items (Study 2: *n* = 260).

Scenario Number and Choice	Mean	*SD*	Skewness	Kurtosis	EFA Factor Loading	
					Original	Final
01A	2.51	1.31	−0.48	−0.93	0.53	0.53
02B	1.60	1.15	0.31	−0.75	0.36	0.34
03E	1.80	1.04	0.04	−0.39	0.31	---
04A	2.79	1.11	−0.78	−0.16	0.65	0.66
05C	1.12	0.99	0.48	−0.61	−0.10	---
06C	2.27	1.10	−0.31	−0.53	0.48	0.49
07A	1.56	1.16	0.21	−0.87	0.28	---
08A	2.58	1.11	−0.62	−0.27	0.55	0.55
09B	2.21	1.04	−0.25	−0.38	0.55	0.55
10D	2.53	1.11	−0.47	−0.35	0.67	0.67
11B	2.03	1.02	−0.22	−0.50	0.50	0.50
12B	2.04	0.98	−0.00	−0.50	0.42	0.41
13B	2.52	1.11	−0.58	−0.31	0.67	0.67
14A	2.21	1.00	−0.44	−0.13	0.61	0.61
15A	2.58	1.14	−0.46	−0.51	0.71	0.71
16C	1.76	1.17	0.11	−0.82	0.34	0.33

Note. KMO = 0.855; Bartlett’s test of sphericity, *χ^2^*(*df*) = 1097.719 (120) (*p* < 0.001) by original items. KMO = 0.876; Bartlett’s test of sphericity, *χ^2^*(*df*) = 903.130 (82) (*p* < 0.001) by final items.

**Table 10:** Mean, SD, skewness, kurtosis, and factor loading of the original and final EFA of TOSCA-3 guilt domain items (Study 2: *n* = 260).

Scenario Number and Choice	Mean	*SD*	Skewness	Kurtosis	EFA	
					Original	Final
01C	3.07	1.14	−1.09	0.30	0.67	0.66
02A	2.76	1.13	−0.69	−0.24	0.61	0.61
03A	2.20	1.10	−0.22	−0.51	0.27	---
04C	2.41	1.04	−0.34	−0.43	0.57	0.57
05D	2.64	1.04	−0.39	−0.55	0.64	0.64
06B	1.97	1.06	−0.18	−0.64	0.32	---
07D	2.81	1.09	−0.76	−0.01	0.68	0.67
08C	2.94	1.01	−0.84	0.31	0.75	0.75
09D	2.71	1.05	−0.68	0.06	0.66	0.67
10C	2.73	1.11	−0.61	−0.38	0.73	0.73
11E	1.72	1.02	0.07	−0.45	0.37	0.37
12D	2.10	1.00	−0.15	−0.10	0.46	0.46
13C	2.67	1.03	−0.48	−0.17	0.73	0.74
14C	2.07	0.92	−0.13	−0.20	0.39	0.39
15C	2.79	1.06	−0.63	−0.30	0.64	0.64
16B	2.57	1.09	−0.42	−0.55	0.67	0.67

Note. KMO = 0.921; Bartlett’s test of sphericity, *χ^2^*(*df*) = 1464.211 (120) (*p* < 0.001) by original items. KMO = 0.933; Bartlett’s test of sphericity, *χ^2^*(*df*) = 1368.140 (91) (*p* < 0.001) by final items.

**Table 11:** Mean, SD, skewness, kurtosis, and factor loading of the original and final EFA of TOSCA-3 alpha-pride domain items (Study 2: *n* = 260).

Scenario Number and Choice	Mean	*SD*	Skewness	Kurtosis	EFA	
					Original	Final
03B	1.87	1.07	−0.15	−0.68	0.54	0.54
06A	1.99	1.09	−0.16	−0.76	0.55	0.55
08D	1.49	1.00	0.12	−0.46	0.37	0.37
11D	1.91	1.03	−0.10	−0.39	0.63	0.63
14E	2.07	0.91	−0.19	−0.00	0.49	0.49

Note. KMO = 0.730; Bartlett’s test of sphericity, *χ^2^*(*df*) = 144.569 (10) (*p* < 0.001).

**Table 12:** Mean, SD, skewness, kurtosis, and factor loading of the original and final EFA of TOSCA-3 beta-pride domain items (Study 2: *n* = 260).

Scenario Number and Choice	Mean	*SD*	Skewness	Kurtosis	EFA	
					Original	Final
03C	2.15	1.11	−0.44	−0.54	0.52	0.52
06D	1.77	0.97	0.01	−0.53	0.51	0.46
08E	1.36	1.04	0.22	−0.62	0.25	---
11C	2.22	1.03	−0.32	−0.37	0.61	0.62
14D	2.47	0.96	−0.51	0.26	0.44	0.47

Note. KMO = 0.635; Bartlett’s Test of Sphericity, *χ^2^*(*df*) = 124.404 (10) (*p* < 0.001) items; KMO = 0.619; Bartlett’s test of sphericity, *χ^2^*(*df)* = 106.104 (16) (*p* < 0.001) by final items.

**Table 13:** Mean, SD, skewness, kurtosis, and factor loading of the original and final EFA of TOSCA-3 externalisation domain items (Study 2: *n* = 260).

Scenario Number and Choice	Mean	*SD*	Skewness	Kurtosis	EFA	
					Original	Final
01D	0.94	1.02	0.80	−1.09	0.65	0.67
02C	1.07	1.06	0.70	−0.15	0.60	0.60
03D	1.78	1.01	−0.01	−0.06	0.34	0.33
04B	1.91	1.15	0.07	−0.41	0.31	---
05A	1.24	1.07	0.31	−0.92	0.59	0.60
06E	1.76	0.92	−0.23	−0.27	0.26	---
07B	1.20	1.07	0.50	−0.10	0.58	0.58
08B	1.14	1.00	0.51	−0.64	0.67	0.68
09A	1.42	1.15	0.30	−0.51	0.45	0.45
10B	1.00	0.95	0.59	−0.27	0.74	0.76
11A	2.15	1.00	−0.27	−0.68	0.04	---
12C	1.36	0.96	0.09	−0.64	0.56	0.54
13A	1.69	1.03	0.11	−1.09	0.42	0.41
14B	1.75	0.91	−0.04	−0.15	0.32	---
15B	1.36	1.12	0.39	−0.60	0.51	0.50
16D	1.11	1.00	0.45	−0.41	0.55	0.55

Note. KMO = 0.846; Bartlett’s Test of Sphericity, *χ^2^*(*df*) = 1035.513 (120) (*p* < 0.001) by original items; KMO = 0.865; Bartlett’s Test of Sphericity, *χ^2^*(*df*) = 871.515 (66) (*p* < 0.001) by final items.

**Table 14:** Mean, SD, skewness, kurtosis, and factor loading of the original and final EFA of TOSCA-3 detached domain items (Study 2: *n* = 260).

Scenario Number and Choice	Mean	*SD*	Skewness	Kurtosis	EFA	
					Original	Final
01B	1.18	1.12	0.60	−0.58	0.49	0.46
02D	1.37	1.02	0.30	−0.58	0.57	0.56
04D	1.66	1.09	0.06	−0.86	0.65	0.65
05B	2.07	1.14	−0.24	−0.63	0.19	---
07C	1.78	1.13	−0.05	−0.95	0.65	0.65
09C	2.21	1.06	−0.33	−0.45	0.48	0.51
10A	1.67	1.13	0.21	−0.76	0.57	0.57
12A	1.95	1.07	−0.36	−0.70	0.39	0.41
13D	2.11	1.03	−0.31	−0.41	0.50	0.52
15D	0.70	0.93	0.90	−0.64	0.26	---
16A	1.27	1.05	0.33	−0.85	0.43	0.40

Note. KMO = 0.817; Bartlett’s Test of Sphericity, *χ^2^*(*df*) = 461.580 36) (*p* < 0.001) by original items; KMO = 0.865; Bartlett’s Test of Sphericity, *χ^2^*(*df*) = 871.515 (66) (*p* < 0.001) by final items.

**Table 15:** Factor structure models of TOSCA-3 self-conscious emotion domains (Study 2).

Model	χ^2^	*df*	χ^2^/*df*	Δχ^2^ (*df*)	CFA	ΔCFI	RMSEA	ΔRMSEA	AIC
Shame									
1-factor	154.359	65	2.735	Ref	0.894	Ref	0.073	Ref	232.359
With method factor	106.286	53	2.055	53.073 (12) ***	0.937	0.043	0.062	0.011	208.286
Guilt									
1-factor	153.052	77	1.988	Ref	0.942	Ref	0.062	Ref	237.052
With method factor	118.244	65	1.819	34.808 (12) ***	0.959	0.017	0.056	0.006	226.244
Alpha-pride									
1-factor	7.4740	5	1.494	Ref	0.982	Ref	0.044	Ref	37.470
Beta-pride									
1-factor	14.176	2	7.088	Ref	0.879	Ref	0.153	Ref	38.176
Externalisation									
1-factor	143.747	54	2.662	Ref	0.891	Ref	0.080	Ref	215.747
With method factor	96.364	45	2.141	47.383 (9) ***	0.938	0.047	0.066	0.014	186.304
Detachment									
1-factor	78.345	27	2.902	Ref	0.881	Ref	0.086	Ref	132.345

Note. *** *p* < 0.001.

**Table 16:** Omega indices for shame, guilt, and externalisation (Study 2).

	ECV	PUC	*ω*/*ω_S_*	*ω_H_*/*ω_HS_*
Shame		0.462		
General factor	0.746		0.869	0.850
Negative scenario factor			0.813	0.000
Positive scenario factor			0.727	0.140
Guilt		0.330		
General factor	0.834		0.928	0.915
Negative scenario factor			0.913	0.003
Positive scenario factor			0.615	0.162
Externalisation		0.303		
General factor	0.689		0.875	0.827
Negative scenario factor			0.846	0.036
Positive scenario factor			0.793	0.424

Note. ECV, explained common variance; PUC, per cent of uncontaminated correlations; ω, coefficient omega; *ωS*, omega subscale; *ωH*, omega hierarchical; *ωHS*, omega hierarchical subscale.

## Data Availability

All the data used in this study are available upon reasonable request to the senior author.

## Supplementary Materials

The following supporting information can be downloaded at: https://www.mdpi.com/article/10.3390/bs14070576/s1. Table S1: Demographics of those participating in Study 2. (*n* = 260).

## References

[1] Ekman P., Friesen W.V. (1969). The repertoire of nonverbal behavior: Categories, origins, usage, and coding. Semiotica.

[2] Plutchik R. (1962). The Emotions: Facts, Theories, and a New Model.

[3] Russell J.A., Mehrabian A. (1977). Evidence for a three-factor theory of emotions. J. Res. Personal..

[4] Tomkins S.S., Arnold M.B. (1970). Affect as the primary motivational system. Feelings and Emotions: The Loyola Symposium.

[5] Kleinginna P.R., Kleinginna A.M. (1981). A categorized list of emotion definitions, with suggestions for a consensual definition. Motiv. Emot..

[6] Ekman P., Friesen W.V. (1975). Unmasking the Face: A Guide to Recognizing Emotions from Facial Clues.

[7] Lazarus R.S. (1991). Emotion and Adaptation.

[8] Panksepp J. (1992). A critical role for “affective neuroscience” in resolving what is basic about basic emotions. Psychol. Rev..

[9] Tracy J.L., Robins R.W. (2004). Putting the self into self-conscious emotions: A theoretical model. Psychol. Inq..

[10] Tracy J.L., Robins R.W., Tracy J.L., Robins R.W., Tangney J.P. (2007). The self in self-conscious emotions: A cognitive appraisal approach. The Self-Conscious Emotions: Theory and Research.

[11] Buss A.H. (2001). Psychological Dimensions of the Self.

[12] Lewis M., Sullivan M.W., Stanger C., Weiss M. (1989). Self development and self-conscious emotions. Child Dev..

[13] Tangney J.P., Dearing R.L. (2002). Shame and Guilt.

[14] Tangney J.P. (1990). Assessing individual differences in proneness to shame and guilt: Development of the Self-Conscious Affect and Attribution Inventory. J. Personal. Soc. Psychol..

[15] Tangney J.P. (1991). Moral affect: The good, the bad, and the ugly. J. Personal. Soc. Psychol..

[16] Tangney J.P., Wagner P., Gramzow R. (1992). Proneness to shame, proneness to guilt, and psychopathology. J. Abnorm. Psychol..

[17] Tangney J.P., Stuewig J., Mashek D.J. (2007). Moral emotions and moral behavior. Annu. Rev. Psychol..

[18] Tracy J.L., Mercadante E., Hohm I. (2023). Pride: The emotional foundation of social rank attainment. Annu. Rev. Psychol..

[19] Gao J., Qin M., Qian M., Liu X. (2013). Validation of the TOSCA-3 among Chinese young adults. Soc. Behav. Personal. Int. J..

[20] Gouva M., Kaltsouda A., Paschou A. (2012). Psychometric Evaluation of the Greek version of TOSCA-3 to measure Shame and Guilt. Intersci. Health Care.

[21] Lacerenza C.N., Joseph D.L., Cassisi J.E. (2020). Are we assessing guilt correctly? An investigation of the psy-chometric properties of a prominent guilt measure. Motiv. Emot..

[22] Eterović M., Medved V., Bilić V., Kozarić-Kovačić D., Žarković N. (2019). Poor agreement between two commonly used measures of shame-and guilt-proneness. J. Personal. Assessment.

[23] Eterović M., Marinić L.M., Medved V., Bilić V. (2020). Croatian version of the Test of self-conscious affect-3: Gender differences and agreement with subjectively perceived shame-and guilt-proneness. Curr. Psychol..

[24] Tangney J.P., Miller R.S., Flicker L., Barlow D.H. (1996). Are shame, guilt, and embarrassment distinct emotions?. J. Personal. Soc. Psychol..

[25] Tangney J.P., Tangney J.P., Fischer K.W. (1995). Shame and guilt in interpersonal relationships. Self-Conscious Emotions: The Psychology of Shame, Guilt, Embarrassment, and Pride.

[26] Podsakoff P.M., MacKenzie S.B., Lee J.-Y., Podsakoff N.P. (2003). Common method biases in behavioural research: A critical review of the literature and recommended remedies. J. Appl. Psychol..

[27] Steinmann I., Strietholt R., Braeken J. (2022). A constrained factor mixture analysis model for consistent and inconsistent respondents to mixed-worded scales. Psychol. Methods.

[28] Xin Z., Chi L. (2010). Wording effect leads to a controversy over the construct of the Social Dominance Orientation Scale. J. Psychol..

[29] Schmitt N., Stults D.M. (1985). Factors defined by negatively keyed items: The result of careless respondents?. Appl. Psychol. Meas..

[30] Woods C.M. (2006). Careless responding to reverse-worded items: Implications for confirmatory factor analysis. J. Psychopathol. Behav. Assess..

[31] Hasui C., Igarashi H., Shikai N., Shono M., Nagata T., Kitamura T. (2009). The Resilience Scale: A duplication study in Japan. Open Fam. Stud. J..

[32] Hiramura H., Shono M., Tanaka N., Nagata T., Kitamura T. (2008). Prospective study on suicidal ideation among Japanese undergraduate students: Correlation with stressful life events, depression, and depressogenic cognitive patterns. Arch. Suicide Res..

[33] Igarashi H., Hasui C., Uji M., Shono M., Nagata T., Chen Z., Kitamura T., Villanueva J.P. (2010). Narcissistic and borderline personality traits: Their relationship with childhood abuse experiences in a student population in Japan. Personality Traits: Classifications, Effects and Changes.

[34] Igarashi H., Hasui C., Uji M., Shono M., Nagata T., Kitamura T. (2010). Effects of child abuse history on borderline personality traits, negative life events, and depression: A study among a university student population in Japan. Psychiatry Res..

[35] Kitamura T., Nagata T. (2014). Suicidal ideation among Japanese undergraduate students: Relationships with borderline personality trait, depressive mood, and childhood abuse experiences. Am. J. Psychol. Behav. Sci..

[36] Liu Q., Nagata T., Shono M., Kitamura T. (2009). The effects of adult attachment style and negative life events on daily depression: A sample of Japanese university students. J. Clin. Psychol..

[37] Sakata M., Takagishi Y., Kitamura T. (2013). Factor structure of the Japanese version of the Coping Inventory for Stressful Situations: Reclassification of coping styles and predictive power for depressive mood. Psychol. Psychother..

[38] Shikai N., Nagata T., Kitamura T. (2014). Do people cope with situations as they say? Relationship between perception of coping styles and actual coping behaviors. Psychiatry Clin. Neurosci..

[39] Shikai N., Uji M., Shono M., Nagata T., Kitamura T. (2008). Dispositional coping styles and childhood abuse history among Japanese undergraduate students. Open Fam. Stud. J..

[40] Takagishi Y., Sakata M., Kitamura T. (2014). Factor structure of the Coping Inventory for Stressful Situations (CISS) in Japanese workers. Psychology.

[41] Uji M., Kitamuta T., Nagata T. (2009). Study on the relationship between the perceived maternal parenting and self-conscious affects. Arch. Int. J. Med..

[42] Yamada F., Kataoka Y., Nagata T., Kitamura T. (2022). Development and validation of a short version of the primary scales of the Inventory of Personality Organization: A study among Japanese university students. Psychology.

[43] Tangney J.P., Dearing R.L., Wagner P.E., Gramzow R. (2000). The Test of Self Conscious Affect-3 (TOSCA-3).

[44] Kitamura T., Suzuki T. (1986). Nihongoban Social Desirability Scale nit suite [On the Japanese version of the Social Desirability Scale]. Jpn. J. Soc. Psychiatry.

[45] Crowne D.P., Marlowe D. (1960). A new scale of social desirability independent of psychopathology. J. Couns. Psychol..

[46] Zung W.W.K. (1965). A self-rating depression scale. Arch. Gen. Psychiatry.

[47] Kitamura T., Hirano H., Chen Z., Hirata M. (2004). Factor structure of the Zung Self-rating Depression Scale in first-year university students in Japan. Psychiatry Res..

[48] Tanno Y., Sakamoto S., Ishigaki T., Sugiura Y., Mouri I. (1998). Yukuutsu to suiron no ayamari: Suiron no ayamari shakudo (TES) no sakusei [depression and logical thinking error: Development of the Thought Error Scale]. J. Konohana Clin. Psychol..

[49] Sakamoto S., Tanaka E., Tanno Y., Ono Y. (2004). Beck no yukuutsumoderu no kentou: DAS to ATQO wo mochiite [Testing Beck’s model of depression; Using the DAS and the ATQ]. Psychol. Res. Nohon Univ..

[50] Kendall P.C., Howard B.L., Hays R.C. (1989). Self-referent speech and psychopathology: The balance of positive and negative thinking. Cogn. Ther. Res..

[51] Hollon S.D., Kendall P.C. (1980). Cognitive self-statements in depression: Development of an automatic thoughts questionnaire. Cogn. Ther. Res..

[52] Chioqueta A.P., Stiles T.C. (2004). Norwegian version of the Automatic Thoughts Questionnaire: A reliability and validity study. Cogn. Behav. Ther..

[53] Sahin N.H., Sahin N. (1992). Reliability and validity of the Turkish version of the Automatic Thoughts Questionnaire. J. Clin. Psychol..

[54] Kwon S., Yoon H. (1994). Construction and utilization of the Korean version of the Automatic Thoughts Questionnaire. Stud. Rev..

[55] Furukawa T., Suzuki-Moor A., Saito Y., Hamanaka T. (1993). Reliability and validity of the Japanese version of the coping inventory for stressful situations (CISS): A contribution to the cross-cultural studies of coping. Seishin Shinkeigaku Zasshi.

[56] Endler N.S., Parker J.D. (1990). Multidimensional assessment of coping: A critical evaluation. J. Personal. Soc. Psychol..

[57] Kitamura T., Suzuki T. (1993). A validation study of Parental Bonding Instrument in Japanese Population. Jpn. J. Psychiatry Neurol..

[58] Kitamura T., Suzuki T. (1993). Perceived rearing attitudes and minor psychiatric morbidity among Japanese adolescents. Jpn. J. Psychiatry Neurol..

[59] Parker G.B., Tupling H., Brown L.B. (1979). A parental and bonding instrument. Br. J. Med. Psychol..

[60] Sanders B., Becker-Lausen E. (1995). The measurement of psychological maltreatment: Early data on the child abuse and trauma scale. Child Abus. Negl..

[61] Wagnild G., Young H. (1993). Development and psychometric evaluation of the Resilience Scale. J. Nurs. Meas..

[62] Narita K., Shimonaka Y., Nakazato K., Kawaai C., Sato S., Osada Y. (1995). Tokuseiteki-jikokouryokukann-shakudo no kenntou: Shougai-haltutatuteki-riyou no kanousei wo saguru [A Japanese version of the generalized self-efficacy scale: Scale utility from the life-span perspective]. Jpn. J. Educ. Psychol..

[63] Sherer M., Maddux J.E., Mercandante B., Printice-Dunn S., Jancob B., Rogers R.W. (1982). The self-efficacy scale: Construction and validation. Psychol. Rep..

[64] Bandura A. (1977). Self-efficacy: Toward a unifying theory of behavioral change. Psychol. Rev..

[65] Bandura A., Schwarzer R. (1992). Exercise of personal agency through the self-efficacy mechanism. Self-Efficacy: Thought Control of Action.

[66] Bandura A. (1995). Self-Efficacy in Changing Societies.

[67] Igarashi H., Kikuchi H., Kano R., Mitoma H., Shono M., Hasui C., Kitamura T. (2009). The Inventory of Personality Organisation: Its psychometric properties among student and clinical populations in Japan. Ann. Gen. Psychiatry.

[68] Lenzenweger M.F., Clarkin J.F., Kernberg O.F., Foelsch P.A. (2001). The Inventory of Personality Organization: Psychometric properties, factor composition, and criterion relations with affect aggressive dyscontrol, psychosis proneness, and self-domains in a nonclinical sample. Psychol. Assess..

[69] Kernberg O.F. (1975). Borderline Conditions and Pathological Narcissism.

[70] Oashi O. (2004). Relation of type A behavior and multidimensionally measured narcissistic personality of Japanese university students. Psychol. Rep..

[71] Raskin R.N., Hall C.S. (1979). A narcissistic personality inventory. Psychol. Rep..

[72] Emmons R.A. (1984). Factor analysis and construct validity of the narcissistic personality inventory. J. Personal. Assess..

[73] Oshio A. (2004). Jikoai no Seinen Shinrigaku The Adolescent Psychology of Narcissism.

[74] Matsuoka N., Uji M., Hiramura H., Chen Z., Shikai N., Kishida Y., Kitamura T. (2006). Adolescents’ attachment style and early experiences: A gender difference. Arch. Women’s Ment. Health.

[75] Bartholomew K., Horowiz L.M. (1991). Attachment styles among young adults: A test of four-category model. J. Personal. Soc. Psychol..

[76] Griffin D.W., Bartholomew K., Bartholomew K., Perlman D. (1994). The metaphysics of measurement: The case of adult attachment. Advances in Personality Relationship vol. 5 Attachment Processes in Adulthood.

[77] Burton L.J., Mazerolle S.M. (2011). Survey instrument validity Part I: Principles of survey instrument development and validity in athletic training education research. Athl. Train. Educ. J..

[78] Costello A.B., Osborne J.W. (2005). Best practices in exploratory factor analysis: Four recommendations for getting the most from your analysis. Pract. Assess. Res. Eval..

[79] Bentler P.M. (1990). Comparative fit indexes in structural models. Psychol. Bull..

[80] Schermelleh-Engel K., Moosbrugger H., Müller H.H. (2003). Evaluating the fit of structural equation models: Tests of significance and descriptive goodness-of-fit measures. Methods Psychol. Res. Online.

[81] Reise S.P. (2013). The rediscovery of bifactor measurement models. Multivar. Behav. Res..

[82] Reise S.P., Morizot J., Hays R.D. (2007). The role of the bifactor model in resolving dimensionality issues in health outcome measures. Qual. Life Res..

[83] Bonifay W.E., Reise S.P., Scheines R., Meijer R.R. (2015). When are multidimensional data unidimensional enough for structural equation modeling? An evaluation of the DETECT multidimensionality index. Struct. Equ. Model. A Multidiscip. J..

[84] Reise S.P., Moore T.M., Haviland M.G. (2010). Bifactor models and rotations: Exploring the extent to which multidimensional data yield univocal scale scores. J. Personal. Assess..

[85] Reise S.P., Bonifay W.E., Haviland M.G. (2013). Scoring and modeling psychological measures in the presence of multidimensionality. J. Personal. Assess..

[86] Rodriguez A., Reise S.P., Haviland M.G. (2016). Applying bifactor statistical indices in the evaluation of psychological measures. J. Personal. Assess..

[87] Jarrett R.B., Weissenburger J.E. (1990). Guilt in depressed outpatients. J. Consult. Clin. Psychol..

[88] O’Connor L.E., Berry J.W., Weiss J., Gilbert P. (2002). Guilt, fear, submission, and empathy in depression. J. Affect. Disord..

[89] Prosen M., Clark D.C., Harrow M., Fawcett J. (1983). Guilt and conscience in major depressive disorders. Am. J. Psychiatry.

[90] Quiles Z.N., Bybee J. (1997). Chronic and predispositional guilt: Relations to mental health, prosocial behaviour, and religiosity. J. Personal. Assess..

[91] Stompe T., Ortwein-Swoboda G., Chaudhry H.R., Friedmann A., Wentzel T., Schanda H. (2002). Guilt and depression: A cross-cultural comparative study. Psychopathology.

[92] Harder D.W., Cutler L., Rockart L. (1992). Assessment of shame and guilt and their relationships to psychopathology. J. Personal. Assess..

[93] O’Connor L.E., Berry J.W., Weiss J. (1999). Interpersonal guilt, shame, and psychological problems. J. Soc. Clin. Psychol..

[94] Bennett D.S., Sullivan M.W., Lewis M. (2005). Young children’s adjustment as a function of maltreatment, shame, and anger. Child Maltreatment.

[95] Stuewig J., McCloskey L.A. (2005). The relation of child maltreatment to shame and guilt among adolescents: Psychological routes to depression and delinquency. Child Maltreatment.

[96] Tracy J.L., Cheng J.T., Martens J.P., Robins R.W. (2011). The emotional dynamics of narcissism: Inflated by pride, deflated by shame. The Handbook of Narcissism and Narcissistic Personality Disorder: Theoretical Approaches, Empirical Findings, and Treatments.

[97] Hada A., Ohashi Y., Usui Y., Kitamura T. (2023). A scale of parent-to-child emotions: Adaptation, factor structure, and measurement invariance. Fam. Process.

[98] Boiger M., Mesquita B., Uchida Y., Feldman Barrett L. (2013). Condoned or condemned: The situational affordance of anger and shame in the United States and Japan. Personal. Soc. Psychol. Bull..

[99] Lebra T.S. (1983). Shame and guilt: A Psychocultural view of the Japanese self. Ethos.

[100] Bedford O., Hwang K.K. (2003). Guilt and shame in Chinese culture: A cross-cultural framework from the perspective of morality and identity. J. Theory Soc. Behav..

[101] Tangney J.P. (1998). How does guilt differ from shame?. Guilt and Children.

[102] Rothbaum F., Rosen K., Ujiie T., Uchida N. (2002). Family systems theory, attachment theory, and culture. Fam. Process.

