# Supplementary material for: Development of the Japanese Version of the Test of Self-Conscious Affect-3 (TOSCA-3): A Study among Student and Parent Population
Source: Behav Sci (Basel). 2024 Jul 7;14(7):576. doi: 10.3390/bs14070576 (PMC11273926; doi:10.3390/bs14070576)
Supplement: Supplementary file 1 [file behavsci-14-00576-s001.zip › behavsci-3015104-supplementary.pdf]

Supplementary Table S1. Demographics of those participating in Study 2. (n = 260)

	frequency	%
Gender		
Men	130	50
Women	130	50
Marital status		
Married	252	96.9
Unmarried	8	3.1
Education		
Secondary school	5	1.9
High school	48	18.5
Junior college or vocational school	54	20.8
Bachelor's	121	46.5
Master's	23	8.8
Doctorate	3	1.2
Others	6	2.3
Occupation		
Manager/Executive	4	1.5
Company employee	99	38.1
Contractor/Temporary worker	2	0.8
Part-time job	30	11.5
Civil servant (excluding teaching staff)	27	10.4
Self-employed/Free-enterprise	5	1.9
Housewife/Househusband	56	21.5
Student	1	0.4
Professional (Chartered accountant, Lawyer, Tax accountant, Judicial scrivener)	12	4.6
Other	2	0.8
